# Co-Removal of Fe/V Impurity in H_2_TiO_3_ Synthesized from Ti-Bearing Blast Furnace Slag

**DOI:** 10.3390/nano14010012

**Published:** 2023-12-20

**Authors:** Fan Yang, Qiugui Peng, Jing Wang, Lan Xiang

**Affiliations:** 1 Department of Chemical Engineering, Tsinghua University, Beijing 100084, China; yangfan20@mails.tsinghua.edu.cn; 2 School of Chemical Engineering & Pharmacy, Wuhan Institute of Technology, Wuhan 430205, China; pqiugui@yeah.net; 3 Key Laboratory of Synthetic and Biological Colloids, Ministry of Education, School of Chemical and Material Engineering, Jiangnan University, Wuxi 214122, China

**Keywords:** Ti-bearing blast furnace slag, TiO_2_, impurity removal, thermodynamic calculations, hydrothermal hydrolysis

## Abstract

Ti-bearing blast furnace slag (TBFS) can be converted to impurity bearing TiOSO_4_ solution for TiO_2_ pigment production. However, the H_2_TiO_3_ (MTA) hydrolyzed from the solution has too high Fe/V impurity to meet the standard for TiO_2_ pigment. In this study, we found that Fe^3+^ and V^3+^ were easily hydrolyzed and entered the MTA lattice, and hence could not be removed by washing. Furthermore, Fe/V was hard to co-remove by the traditional reduction method. Therefore, the Fe/V non-hydrolysis condition (Ti^3+^ = 0.01 M, *F* = 3.0, T = 130 °C; Ti^3+^ = 0.01 M, *F* = 3.5, T = 150 °C) was determined by thermodynamic calculations. However, at these conditions, the Ti hydrolysis ratio was low or the reaction time was long. Therefore, a new two-step hydrothermal hydrolysis process was proposed. Step 1 (130 °C, 2 h) ensured the non-hydrolysis of V^3+^, and Ti was partially hydrolyzed to increase the H_2_SO_4_ concentration. Step 2 (150 °C, 2 h) ensured a high Ti hydrolysis ratio (>0.95) and short total reaction time (4–6 h). Finally, a high-purity MTA was obtained (Fe = 21 ppm, V = 145 ppm). These results provide new insights into the control of the hydrolysis of impurity ions in solutions and help to optimize the process of TiO_2_ pigment preparation from TBFS.

## 1. Introduction

Titanium-bearing blast furnace slag (TBFS) is a byproduct during the smelting of vanadium-bearing titanomagnetite, which contains up to 23 wt.% TiO_2_ and various impurities (Ca, Si, Al, Mg, Fe, V, Mn) [1,2]. In China, TBFS production is more than 5 million t/a. The high content of TiO_2_ reduces the CaO activity, which inhibits applications in cement production, resulting in a low utilization rate (<3%) and significant slag accumulation (80–100 million tons in total) [2,3].

Extraction of valuable elements by hydrometallurgy has received widespread attention due to its low cost [4,5,6]. Recently, the extraction of Ti from TBFS by the sulfuric acid roasting method has received wide attention because of its energy saving, low cost, and high Ti recovery rate [7,8,9,10]. In this method, TBFS is roasted with concentrated H_2_SO_4_ (80–95%) at 100–300 °C, then leached and filtered to form an impurities-bearing TiOSO_4_ solution, where the extraction of Ti is ~88% [7,11]. Like the sulfate process for TiO_2_ pigment production, the impurities-bearing TiOSO_4_ solution hydrolyzes to form H_2_TiO_3_ (MTA). Then MTA is calcined to form TiO_2_ pigment [11,12]. However, due to the low concentration of TiOSO_4_ (0.6–0.8 mol·L^−1^, vs. 2.4–2.8 mol·L^−1^ in the industry) and the various impurities (Al, Mg, Fe, V, Mn), the production of pigment-grade TiO_2_ has not at this time been achieved [9,11,12].

One important reason is that some impurity ions hydrolyze at high temperatures and co-precipitate with MTA, which cannot be removed by washing [13,14]. The color-developing impurity elements in the impurities-bearing TiOSO_4_ solution include Mn, Fe, and V, which all lower the whiteness of pigment [15]. The occurrence of these elements is as the dissolved species Mn^2+^, Fe^3+^/Fe^2+^, and VO^2+^/V^3+^, respectively. Among them, Fe^3+^ and V^3+^ are easily hydrolyzed [12,16,17]. However, there are strict requirements for the content of both Fe (30 ppm) and V (7 ppm) in MTA [12,18].

For the removal of Fe and V from MTA, the method used in the sulfate process is to reduce Fe^3+^ to Fe^2+^ and use high concentrations of H_2_SO_4_ to inhibit the hydrolysis of V^3+^ [12,18]. However, the impurities-bearing TiOSO_4_ solution from TBFS has a low concentration of H_2_SO_4_ and more V^3+^ [11], so the V^3+^ can more readily hydrolyze. If V^3+^ is oxidized to VO^2+^, this will lead to the formation and possible hydrolysis of Fe^3+^, so it is difficult to achieve acceptable co-removal of Fe/V. Therefore, it is necessary to increase the H_2_SO_4_ concentration by vacuum evaporation, which significantly increases the energy consumption [19,20]. Another viable option is to separate the Fe/V from the original solution, such as via adsorption, extraction, etc. Elnagar et al. [21] extracted trace Fe^3+^ (0.03–80 mg/L) from titanium concentrates with organic complexation and solvent extraction (efficiency > 98.50%). Middlemas et al. [22] proposed a new method for the production of titanium dioxide pigment, which used solvent extraction to remove Fe^3+^ and finally produce good quality anatase pigment (Fe < 20 ppm). Peng et al. [23] adsorbed vanadium(V) with melamine, resulting in 99.89% removal. However, these methods have high agent consumption, are long processes, and have increased costs.

In this study, for the MTA made from impurity bearing TiOSO_4_ solution, we found the Fe/V impurity could not be co-removed by the acid neutralization followed by solution reduction method traditionally. Therefore, we determined the conditions for non-hydrolysis of Fe/V by thermodynamic calculation. However, at these conditions, the Ti hydrolysis ratio was low and the reaction time was long. Therefore, we provided a new simplified two-step hydrolysis process. Without acid neutralization, Ti^3+^ in the original solution inhibited the hydrolysis of Fe. Step 1 (130 °C, 2 h) ensured the non-hydrolysis of V and generated enough free H_2_SO_4_. Step 2 (150 °C, 2 h) promoted the hydrolysis of TiOSO_4_. Finally, high purity MTA was obtained (Fe = 21 ppm, V = 145 ppm) with a high Ti hydrolysis ratio (>0.95) and reasonable reaction time (4–6 h).

## 2. Materials and Methods

### 2.1. Materials and Reagents

TBFS was obtained from the Panzhihua Iron & Steel (Group) Co., Ltd. (Panzhihua, China). The TBFS composition determined by X-ray fluorescence spectroscopy (XRF) is shown in Table 1. Commercial chemicals of analytical grade and deionized water with a resistivity >18 MΩ·cm^−1^ were used in the experiments. Titanyl sulfate-sulfuric acid hydrate (TiOSO_4_·*x*H_2_O·*y*H_2_SO_4_, 93%), aluminum sulfate octa decahydrate (Al_2_(SO_4_)_3_·18H_2_O, 99%), and anhydrous magnesium sulfate (MgSO_4_, 99%) were purchased from Shanghai Macklin Biochemical Co., Ltd. (Shanghai, China). Ammonium iron (III) sulfate dodecahydrate (NH_4_FeSO_4_·12H_2_O, 99%), calcium oxide (CaO, 99%), methyl orange (indicator grade), and ammonium thiocyanate (NH_4_SCN, 99%) were purchased from Shanghai Macklin Biochemical Co., Ltd. (Shanghai, China). Sulfuric acid (H_2_SO_4_, 98%), aluminum powder (Al, 99%), and sodium hydroxide (NaOH, 99%) were purchased from Beijing Tong Guang Fine Chemicals Company (Beijing, China). All of the chemicals were used without any further purification.

### 2.2. Experimental Method

The process of making impurity bearing TiOSO_4_ solution from TBFS was according to our previous research [7,11,24]: TBFS was decomposed by 90% H_2_SO_4_ at 150 °C for 1 h to obtain solid mixtures. The mixtures were leached with water at 65 °C for 1 h and filtered to separate the solid (mainly SiO_2_ and CaSO_4_) to obtain the impurity bearing TiOSO_4_ solution. The impurities were sulfates of various impurity ions. The properties of TBFS and the reactions of the TBFS decomposition were studied previously [2,7].

The composition of the solution was determined by using inductively coupled plasma atomic emission spectroscopy (ICP-AES), as shown in Table 2. The *F*-value is the acidity factor determined as given by Equation (1)

(1) $$F=\frac{m\left(H_{2}SO_{4}\;in\;TiOSO_{4}\right)+m\left(Free\;H_{2}SO_{4}\right)}{m\left(TiO_{2}\right)}$$

where *m* is the mass concentration, g/L. The H_2_SO_4_ in TiOSO_4_ refers to the H_2_SO_4_ generated by the hydrolysis of TiOSO_4_. The sum of the two is referred to as the effective H_2_SO_4_ in the sulfate process [25]. The effective H_2_SO_4_ is determined by 0.5000 mol·L^−1^ NaOH neutralization titration (methyl orange as the indicator) [11]. The concentration of Ti^3+^ was determined by titration with 0.1000 mol·L^−1^ NH_4_FeSO_4_ (NH_4_SCN as the indicator).

The impurity bearing TiOSO_4_ solution was treated with two different processes. Following the traditional sulfate process, the solution was neutralized by CaO to adjust the *F*-value = 1.50, then the Ti^3+^ concentration increased by reacting with Al powder for 1–5 h, and finally thermally hydrolyzed at 110 °C for 4 h at atmospheric pressure. For the new two-step hydrothermal hydrolysis, without any pre-treatment, the solution was sealed in a Teflon-lined autoclave, and firstly hydrothermally hydrolyzed at 110–130 °C for 2–4 h, and then hydrothermally hydrolyzed at 150 °C for 2 h. After hydrolysis, MTA was obtained by filtering. The MTA was washed twice with 5% H_2_SO_4_ and twice with deionized water and then dried in an oven at 105 °C for 4 h. Then, the MTA was dissolved in 98% H_2_SO_4_ at 250 °C for 0.3–0.5 h, and the clarified solution was diluted by deionized water and used to determine the Fe/V content in the MTA by using ICP-AES.

### 2.3. Characterization

The element analysis of the power sample was carried out by X-ray fluorescence spectroscopy (XRF, ARL PERFORM X, Thermo Fisher, Waltham, MA, USA). The solution ion concentration was determined by inductively coupled plasma atomic emission spectroscopy (ICP-AES, ARCOS II, Spectro, Kleve, Germany). The phase was identified from measurements using an X-ray diffractometer (Bruker-AXS D8 Advance, Karlsruhe, Germany) with Cu Kα (0.154178 nm) radiation. The surface composition of the samples was examined with an X-ray photoelectron spectrometer (XPS, PHI-5300, PHI, Lafayette, LA, USA) using an Al Kα (1486.6 eV) X-ray source. All spectra were calibrated to the binding energy of the adventitious C 1 s peak at 284.8 eV. The morphology and microstructure of the samples were characterized with a field emission scanning electron microscope (FESEM, JSM 7401F, JEOL, Hitachi, Tokyo, Japan).

## 3. Results and Discussion

### 3.1. The Hydrolysis Behavior and Occurrence form of Fe/V in MTA

The main impurity elements of TiOSO_4_ solution obtained from TBFS were Mg, Al, Fe, V, and Mn, where both Fe and V possibly existed in two states (Fe^2+^/Fe^3+^ and V^3+^/VO^2+^). Table 3 shows the theoretical pH at the beginning of the hydrolysis of these impurity ions. The precipitation pH was calculated by the ion concentrations and *K*_sp_ of the corresponding hydroxides. Al^3+^, Fe^3+^, and V^3+^ had low precipitation pH, which meant they were easy to hydrolysis.

**Figure 1:**
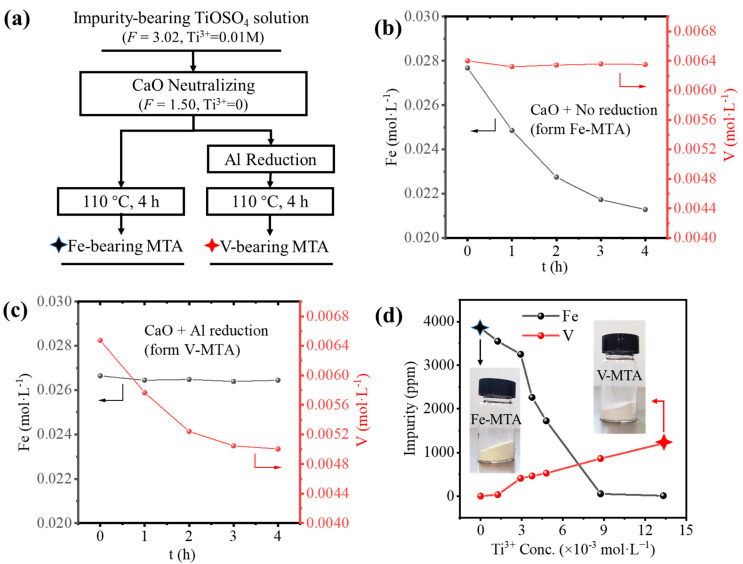
(**a**) The two processes for preparing MTA from the impurity bearing TiOSO_4_ solution. (**b**) The behavior of Fe/V hydrolysis for the formation of Fe-MTA. (**c**) The behavior for Fe/V hydrolysis for the formation of V-MTA. (**d**) Influence of Ti^3+^ concentration on the Fe/V impurity content in MTA. The black star marks the sample Fe-MTA, and the red star marks the V-MTA.

Figure 1a summaries the two processes for the synthesis of MTA from the impurity bearing TiOSO_4_ solution. Firstly, in order to ensure the hydrolysis ratio would be >95%, CaO slurry was added to neutralize the H_2_SO_4_ with a decrease in acidity from *F* = 3.02 to *F* = 1.50 (*F* is the acidity factor defined in Equation (1)). The reduction of the solution acidity promoted the hydrolysis of Fe/V. If the solution was directly hydrolyzed at 110 °C, Fe-MTA was obtained consistent with the hydrolysis of Fe^3+^. In the sulfate process, Ti^3+^ was used to reduce the Fe^3+^ to Fe^2+^ to inhibit the hydrolysis of Fe and the adsorption of Fe^3+^ [12]. In order to generate Ti^3+^, we applied Al reduction ($Al+TiO^{2+}+2H^{+}=Al^{3+}+Ti^{3+}+H_{2}O$). The added Al did not significantly affect the concentration of Al in the solution. However, if the solution was reduced, V-MTA was formed. The composition of Fe-MTA and V-MTA is shown in Table 4; all the impurity contents are in terms of TiO_2_ wt.%. Though Al was hydrolyzed, it was colorless and therefore did not affect the pigment quality.

Figure 1b shows the Fe/V hydrolysis behavior in the unreduced solution. The concentration of V was unchanged, while the concentration of Fe was decreasing exponentially. This means the only Fe was hydrolyzed and precipitated with MTA. With the hydrolysis of TiOSO_4_, the concentration of H_2_SO_4_ was increased ($TiOSO_{4}+{2H}_{2}O=H_{2}TiO_{3}↓+H_{2}SO_{4}$), so finally the hydrolysis of Fe was inhibited and the concentration of Fe was unchanged. Figure 1c shows the Fe/V hydrolysis behavior in the Al reduced solution. Though Fe was unhydrolyzed due to Al reduction with an unchanged concentration, V was hydrolyzed and the concentration was decreasing exponentially like Fe. The reason for this was the formation of V^3+^, as shown in Equations (2)–(4), where *E*^0^ is the standard electrode potential of the reactions at 25 °C.

(2) $$Fe^{3+}+e^{-}=Fe^{2+}\;E^{0}=0.771V\;$$

(3) $$VO^{2+}+2H^{+}+e^{-}=V^{3+}+H_{2}O\;E^{0}=0.337V$$

(4) $$TiO^{2+}+2H^{+}+e^{-}=Ti^{3+}+H_{2}O\;E^{0}=0.100V$$

When the solution was unreduced, no Ti^3+^ was formed, so the solution was in the oxidized state. Fe existed as Fe^3+^, and V existed as VO^2+^. Fe^3+^ was easy to hydrolyze and formed Fe-MTA. When the solution was reduced by Al, Ti^3+^ was generated, Fe^3+^ converted to Fe^2+^, and VO^2+^ converted to V^3+^. V^3+^ was easy to hydrolyze and formed V-MTA. According to the Nernst equation, the concentration of Ti^3+^ affects the redox potential of the solution and finally determines the concentration of Fe^2+^/Fe^3+^ and V^3+^/VO^2+^. Therefore, the aim was to find a suitable Ti^3+^ concentration in the solution for minimal Fe/V hydrolysis, as shown in Figure 1d. The results show that Fe or V in MTA was always >500 ppm at different Ti^3+^ concentrations, so the Fe/V could not be removed simultaneously by Al reduction.

**Figure 2:**
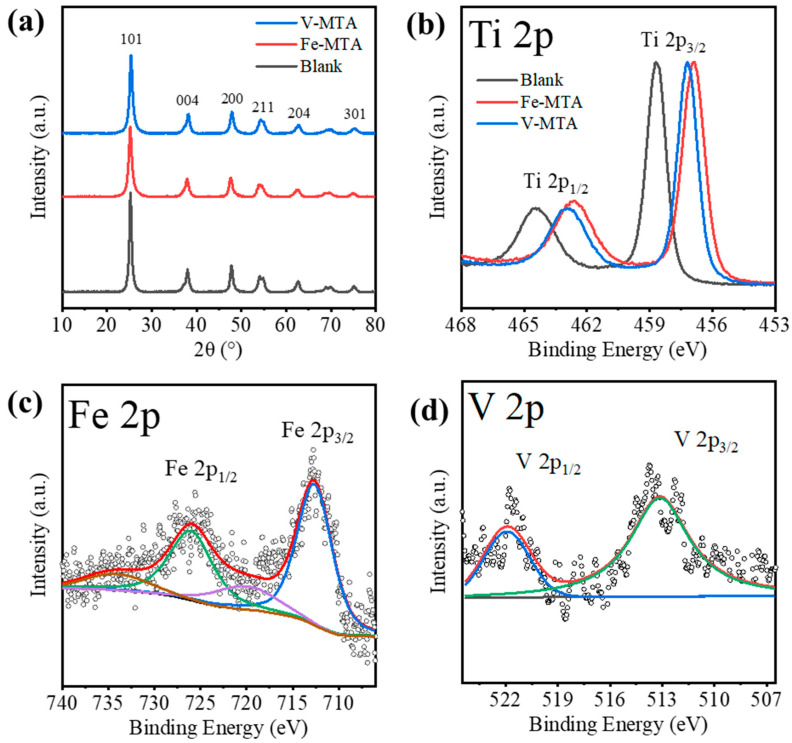
(**a**) XRD patterns and (**b**) XPS Ti 2p spectra of V/Fe/Blank-MTA. (**c**) XPS Fe 2p spectra of Fe-MTA. (**d**) XPS V 2p spectra of V-MTA.

Figure 2a shows the XRD patterns of Fe/V-MTA and blank MTA. The blank MTA was synthesized from the Mg/Al-bearing TiOSO_4_ solution at the same conditions with Fe-MTA. The solution was prepared from reagents (TiOSO_4_, MgSO_4_, Al_2_(SO_4_)_3_) without Fe/V impurities. The crystalline product in these samples was anatase (PDF 21-1272). Table 5 shows the analysis of the (101) peak. Fe/V-MTA had lower diffraction intensity and smaller crystal size than blank MTA, which means the Fe/V in the solution affected the hydrolysis of TiO^2+^ and inhibited the growth of the anatase nanocrystals in MTA. The changes of 2θ for Fe/V-MTA indicate that Fe/V may have entered the anatase nanocrystals and changed the lattice spacing. Figure 2b shows the XPS Ti 2p spectra of Fe/V-MTA and blank MTA. The peaks at ~465.1 and 459.4 eV of blank MTA were assigned to Ti 2p_1/2_ and Ti 2p_3/2_ of the Ti^4+^ state [26]. For Fe/V-MTA, the negative shift of binding energy ~1.8 eV indicated that some Ti^4+^ ions were replaced by Fe/V, and the Ti-O-Ti in anatase changed to Ti-O-Fe/V. Figure 2c shows the XPS Fe 2p spectra of Fe-MTA. The peaks centered at ~723.9 and 712.6 eV were assigned to Fe 2p_1/2_ and Fe 2p_3/2_ of the Fe (III) species, and the spin–orbit splitting characteristic of Fe^3+^ corresponded to typical features of Fe(OH)_3_ [27]. Figure 2d shows the XPS V 2p spectra of V-MTA. The peaks centered at ~521.8 and 514.4 eV were assigned to V 2p_1/2_ and V 2p_3/2_ of V^3+^, which was close to V(OH)_3_ [28,29]. Therefore, the results indicate that some of the Fe^3+^/V^3+^ hydrolyzed with TiO^2+^ at high temperature and entered the lattice of anatase nanocrystals so these impurities could not be removed by dilute H_2_SO_4_ or water.

### 3.2. Thermodynamic Equilibrium Calculation of the Hydrolysis Process

It has been proposed that Fe/V enters the MTA lattice by hydrolysis with TiO^2+^, so the key to controlling the impurity content is to inhibit the hydrolysis of Fe^3+^/V^3+^. It is known that if a reaction cannot proceed thermodynamically, then kinetically the reaction cannot proceed. Therefore, assessing if the Fe^3+^/V^3+^ hydrolysis reaction will happen thermodynamically at different reaction conditions will help to identify the Fe^3+^/V^3+^ non-hydrolysis condition. Therefore, a thermodynamic calculation for the hydrolysis process of impurity bearing TiOSO_4_ solution was carried out. The equilibria of the impurities-bearing TiOSO_4_ solution for the thermodynamic calculation are given in Equations (5)–(16).

(5) $$SO_{4}^{2-}+H^{+}=HSO_{4}^{-}$$

(6) $$HSO_{4}^{-}+H^{+}=H_{2}SO_{4}$$

(7) $$Mg^{2+}+SO_{4}^{2-}=MgSO_{4\left(aq\right)}$$

(8) $$Al^{3+}+SO_{4}^{2-}=AlS{O_{4}}^{+}$$

(9) $$AlS{O_{4}}^{+}+SO_{4}^{2-}=Al(SO_{4}{)}_{2}^{-}$$

(10) $$TiO^{2+}+SO_{4}^{2-}=TiOS{O_{4}}_{\left(aq\right)}$$

(11) $$Fe^{3+}+SO_{4}^{2-}=FeSO_{4}^{+}$$

(12) $$VO^{2+}+SO_{4}^{2-}=VOS{O_{4}}_{\left(aq\right)}$$

(13) $$Fe^{3+}+V^{3+}+H_{2}O=Fe^{2+}+2H^{+}+VO^{2+}$$

(14) $$VO^{2+}+Ti^{3+}=V^{3+}+TiO^{2+}$$

(15) $$2V^{3+}+3SO_{4}^{2-}=V_{2}(SO_{4}{{)}_{3}}_{\left(aq\right)}$$

(16) $$H^{+}+OH^{-}=H_{2}O$$

**Figure 3:**
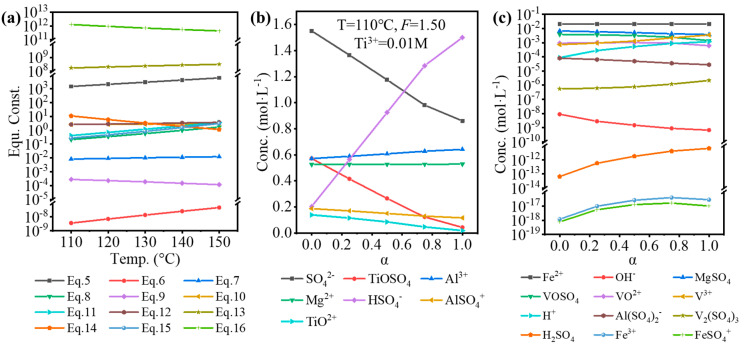
(**a**) Calculated equilibrium constants for Equations (5)–(16) at 110–150 °C. The major (**b**) and minor (**c**) components of the impurities-bearing TiOSO_4_ solution at different hydrolysis ratios (α).

The standard equilibrium constant or relevant electrode potential was obtained from various handbooks and articles [16,17,30,31,32]. By using Van’t Hoff, Kirchhoff’s, and Nernst equations, the equilibrium constants for Equations (5)–(16) at 110–150 °C were obtained, as shown in Figure 3a. By using the Newton–Raphson method, based on the original composition of the solution in Table 2 and equilibrium constants, the equilibrium composition was calculated, as shown in Figure 3b,c. Here, α was referred to as the hydrolysis ratio, which was determined by Equation (17).

(17) $$\alpha =\frac{{\left[TiO^{2+}\right]}_{0}-\left[TiO^{2+}\right]}{{\left[TiO^{2+}\right]}_{0}}$$

The temperature (110 °C), *F*-value (1.50), and [Ti^3+^] (0.01 mol·L^−1^) used in the calculation were the experimental conditions. With increasing hydrolysis ratio, [SO_4_^2−^], [TiOSO_4_], [TiO^2+^], and [OH^−^] decreased, while [HSO_4_^−^], [H^+^], and [H_2_SO_4_] increased, which was because H_2_SO_4_ was formed during the hydrolysis of TiOSO_4_ ($TiOSO_{4}+2H_{2}O=H_{2}TiO_{3}↓+H_{2}SO_{4}$). The concentration of impurity ions changed a little, but it is worth noting that the concentration of H^+^ was relatively low (0.001–0.01 mol·L^−1^). As shown in Table 6, the lower TiOSO_4_ concentration compared to that for the sulfate process contributed to the lower free H_2_SO_4_, corresponding to the lower concentration of H^+^. Even at higher *F*-value, the free H_2_SO_4_ of the impurities-bearing TiOSO_4_ solution was much lower than for the industrial sulfate process in the industry. Furthermore, a large quantity of SO_4_^2-^ introduced by the high concentration of impurity ions from TBFS promoted a positive proceeding of Equation (5), so lots of H^+^ was consumed.

**Figure 4:**
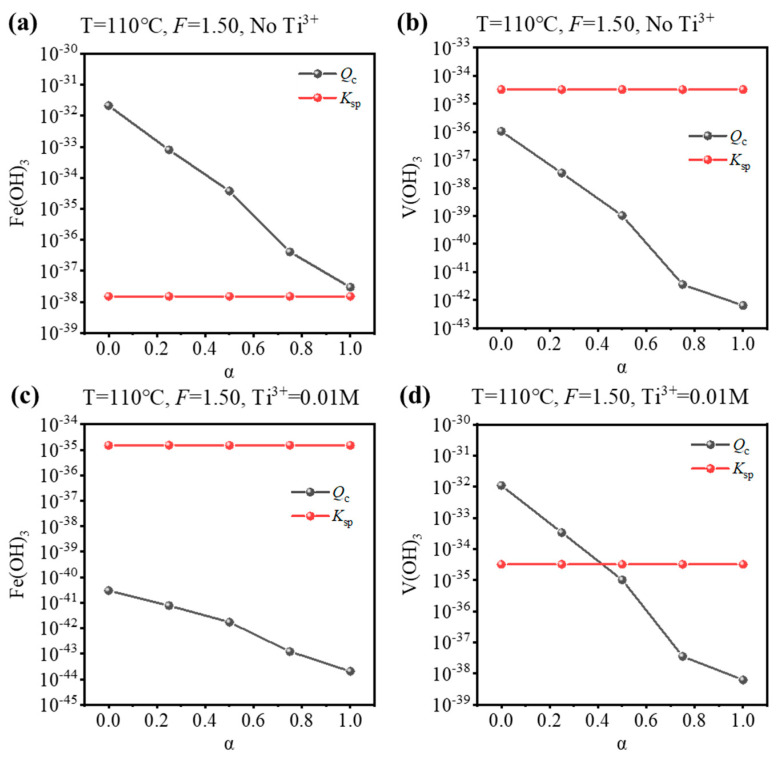
Comparison of the *K*_sp_/*Q*_c_ values for Fe(OH)_3_ and V(OH)_3_ at different hydrolysis ratios (α) under the condition of the synthesis of (**a**,**b**) Fe-MTA (110 °C, *F* = 1.50, No Ti^3+^) and (**c**,**d**) V-MTA (110 °C, *F* = 1.50, 0.01 M Ti^3+^).

Based on the equilibrium composition, the concentration quotient (*Q*_c_) for Fe(OH)_3_ or V(OH)_3_ can be calculated, and whether Fe/V will be precipitated can be judged by comparing it with the corresponding solubility product (*K*_sp_). If *Q*_c_ > *K*_sp_, precipitation is expected, and vice versa. To validate the results of the equilibrium composition, a comparison was performed for the condition that is used to synthesize Fe-MTA and V-MTA, as shown in Figure 4. For the Fe-MTA condition (Figure 4a,b), at any hydrolysis ratio, Fe(OH)_3_ formation was expected as *Q*_c_ > *K*_sp_ and V(OH)_3_ formation was expected as *Q*_c_ < *K*_sp_. For the V-MTA condition (Figure 4c,d), V hydrolyzed while Fe did not at a low hydrolysis ratio, and neither Fe nor V was hydrolyzed at a high hydrolysis ratio. The calculations were consistent with the experimental results. Furthermore, the *Q*_c_ values for Fe(OH)_3_/V(OH)_3_ significantly decreased with the increasing of α, so increasing the concentration of free H_2_SO_4_ (*F*-value) was expected to inhibit the hydrolysis of Fe/V.

**Figure 5:**
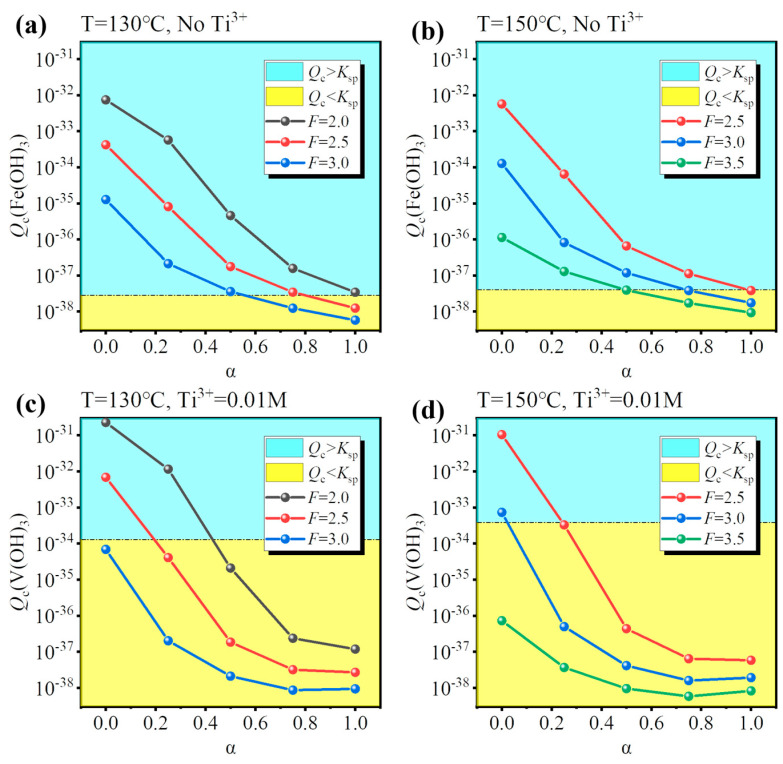
Calculated effect of temperature (130/150 °C) and *F*-value (2.0/2.5/3.0/3.5) for assessing (**a**,**b**) Fe hydrolysis under oxidizing conditions (No Ti^3+^) and (**c**,**d**) V hydrolysis under reducing conditions (0.01 M Ti^3+^).

To identify the conditions for Fe/V co-removal, a simulation for different hydrolysis conditions (temperature, *F*-value, and Ti^3+^ concentration) was performed and the results are shown in Figure 5. As discussed before, increasing the concentration of free H_2_SO_4_ close to that of the industry standard is an effective way to inhibit the hydrolysis of Fe/V, so a suitable *F*-value was 2.0–3.5. However, increasing the *F*-value lowered the hydrolysis ratio, so a higher temperature was necessary (T = 130–150 °C). There were two ways for the co-removal: (1) inhibit Fe hydrolysis under oxidizing conditions (No Ti^3+^) or (2) inhibit V hydrolysis under reducing conditions (0.01 M Ti^3+^). The results show that Fe^3+^ was always hydrolyzed at a low hydrolysis ratio (Figure 5a,b), while V^3+^ had unhydrolyzed conditions (Figure 5c,d). Therefore, method (2) was optimal for Fe/V co-removal. The possible conditions were (130 °C, *F* = 3.0) and (150 °C, *F* = 3.5). However, for the condition (130 °C, *F* = 3.0), the Ti hydrolysis ratio was only 0.638 after 4 h; likewise, for the condition (150 °C, *F* = 3.5), the hydrolysis ratio was 0.842 after 4 h. Therefore, though the two possible conditions may ensure the Fe/V co-removal, they cannot ensure the Ti hydrolysis ratio >0.95 within a reasonable reaction time (4–6 h), which will lower the production efficiency.

### 3.3. Two-Step Hydrothermal Hydrolysis for Fe and V Co-Removal

**Figure 6:**
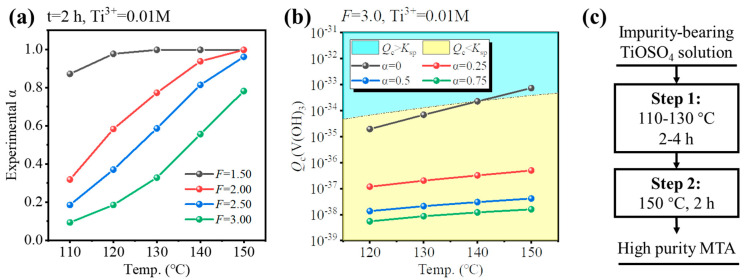
(**a**) Hydrolysis ratio of impurities-bearing TiOSO_4_ solution for 2 h at 110–150 °C and *F* = 1.5–3.0. (**b**) Calculated *Q*_c_ of V(OH)_3_ at *F* = 3.0, 120–150 °C, and various experimental α. (**c**) Concept of two-step hydrothermal hydrolysis process for Fe/V co-removal.

To ensure a high hydrolysis ratio (experimental α > 0.95) within a reasonable reaction time (4–6 h), the hydrolysis properties of impurities-bearing TiOSO_4_ solution were investigated, as shown in Figure 6a. For the solution formed by dissolution of TBFS, the *F*-value was 3.0. If the *F*-value was higher (*F* = 3.5), additional concentrated H_2_SO_4_ was required, and the temperature or reaction time for hydrolysis needed to be higher or longer. If the *F*-value was lower (*F* = 2.5), V^3+^ would hydrolyze during the initial stages of Ti hydrolysis as shown in Figure 5c,d. Thus, *F* = 3.0 was selected as optimal. For *F* = 3.0, the conditions whether V^3+^ hydrolyzed at different temperatures and hydrolysis ratios were investigated, so that several hydrolysis processes were provided as shown in Figure 6a,b. If the solution was hydrolyzed at 150 °C for 4 h, V^3+^ may have hydrolyzed at the initial stages of Ti hydrolysis (*Q*_c_ > *K*_sp_ when α = 0), as shown in Figure 6b. If the solution was hydrolyzed at 130 °C for 4 h, though the V^3+^ was not hydrolyzed, the hydrolysis ratio was very low (0.32, 2 h), as shown in Figure 6a. Therefore, we provided a new two-step hydrothermal hydrolysis method, as shown in Figure 6c. For Step 1, the impurities-bearing TiOSO_4_ solution was hydrolyzed at low temperature to inhibit the V hydrolysis. According to Figure 6b, the suitable condition for hydrolysis was at 110–130 °C for 2–4 h to allow Ti to partially hydrolyze. As hydrolysis proceeded, [H_2_SO_4_] was increased, and the Ti hydrolysis ratio > 0.25, V^3+^ was not hydrolyzed at higher temperatures. Therefore, for Step 2, the solution was hydrolyzed at a higher temperature to ensure α > 0.95 within 6 h. According to Figure 6c, to reduce the total hydrolysis time, the hydrolysis at 150 °C was conducted for 2 h.

**Figure 7:**
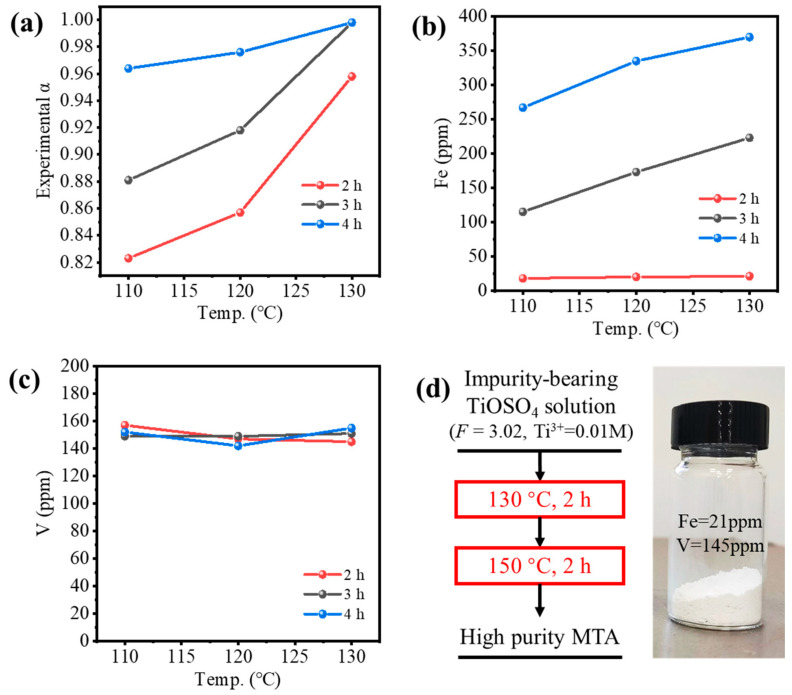
(**a**) Experimental hydrolysis ratio of impurities-bearing TiOSO_4_ solution and (**b**) Fe or (**c**) V content in MTA obtained by the two-step method (different condition for Step 1 in Figure 6c). (**d**) High purity MTA obtained from the optimal two-step process.

Figure 7 shows the experimental validation of the two-step hydrothermal hydrolysis method, where only the conditions of Step 1 were varied. Figure 7a shows the hydrolysis ratio was increased by the temperature and reaction time of Step 1; α > 0.95 could be achieved only if the reaction time was longer than 4 h or the temperature was 130 °C. Figure 7b shows the Fe content in MTA was only 21 ppm for the reaction time of 2 h, which met the requirement of TiO_2_ pigment (<30 ppm) and was much lower than Fe-MTA (3854 ppm), but it increased rapidly with hydrolysis time. This may have been because the oxygen in the autoclave gradually oxidized Ti^3+^ to TiO^2+^ as time went on, while Fe^2+^ was converted to Fe^3+^, resulting in an increase of the Fe content in MTA. Figure 7c shows that the V content in MTA at the different conditions was almost the same, which was in the range of 140–160 ppm. Despite not meeting the requirement of TiO_2_ pigment (>7 ppm), it was much lower than V-MTA (1205 ppm). Considering both the extent of hydrolysis and the Fe/V content, the optimal condition for Step 1 was hydrolysis at 130 °C for 2 h. The sample obtained with high purity is shown in Figure 7d. Compared with the traditional method (Figure 1a), the process was simpler. More importantly, Fe and V were both excluded by the two-step method (Fe = 21 ppm, V = 145 ppm). Compared with the optimal Fe/V content in Figure 1d, the impurities content of the two-step method was notably decreased and the color was white. Compared with previous relevant works [13,14,20], Fe was decreased from ~10,000 ppm to 21 ppm.

**Figure 8:**
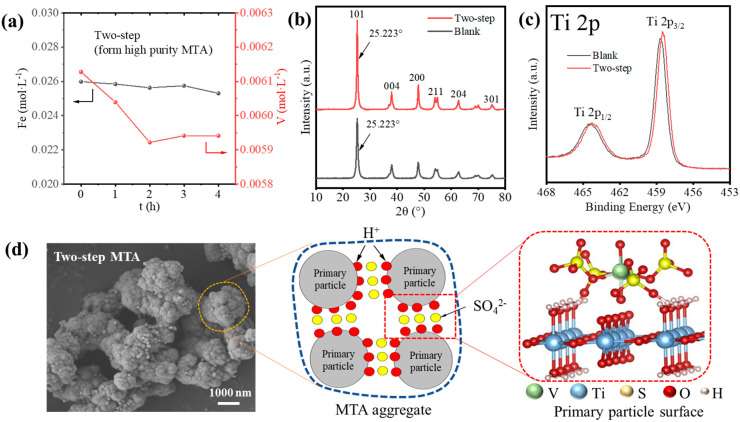
(**a**) The hydrolysis behavior of Fe/V during the formation of two-step MTA. (**b**) XRD patterns and (**c**) XPS Ti 2p spectra of two-step MTA and blank MTA. (**d**) The SEM image and mechanism for how V^3+^ enters the MTA during the two-step hydrolysis.

Though the two-step method reduced the Fe/V content of MTA, the V impurity was still high. Thus, we studied the possible reason for how V^3+^ entered the MTA from which the results are shown in Figure 8. As shown in Figure 8a, the reduction in V concentration up to 4 h was significantly less than before (Figure 1c). Furthermore, Figure 8b shows that the two-step MTA and blank MTA had the same XRD (101) peak position of anatase, which means the V^3+^ did not enter the lattice of MTA. This indicates that V^3+^ did not enter the MTA by hydrolysis but was present due to other mechanisms. Figure 8c compares the Ti 2p XPS spectra of the two-step MTA and blank MTA. The peaks at ~465.1 and 459.4 eV of blank MTA were assigned to Ti 2p_1/2_ and Ti 2p_3/2_ of the Ti^4+^ state. For the two-step MTA, the negative shift of binding energy ~0.2 eV indicated that some V^3+^ was located on the MTA surface and affected the electronic state of Ti^4+^, as previous studies have shown [33]. Therefore, V impurities in MTA may have been due to the adsorption of V^3+^. Furthermore, the V cannot be washed off, so the adsorption was not a simple physical adsorption. According to the formation and structure of MTA, we provided a possible mechanism for how V^3+^ enters the MTA in two-step hydrolysis, as shown in Figure 8d. The SEM image shows that the two-step MTA formed from impurity bearing TiOSO_4_ solution had hierarchical structures [34]; the aggregation of 30–100 nm primary anatase TiO_2_ particles to form final 1–3 μm MTA aggregates. Previous studies show that SO_4_^2−^ (4–6 wt.% SO_3_ of TiO_2_) is strongly adsorbed at the surface of the primary particles [34,35]. Furthermore, V^3+^ can readily complex with SO_4_^2−^, so the V^3+^-SO_4_^2−^ complex may be formed on the primary particles surface of MTA, like the interaction between Al^3+^ and the hydrated TiO_2_ clusters [36,37]. As hydrolysis proceeds, primary particles agglomerate and therefore V^3+^ is trapped in MTA, which is difficult to wash off.

For future studies of Fe/V co-reduced, the key to reduce the V content in MTA is to inhibit the formation of the V^3+^-SO_4_^2−^ complex. Specifically, there are two ways: (1) selective complexation of V^3+^ with complexing agents, and (2) converting V^3+^ to VO^2+^ with oxidizing agents to facilitate the dissolution of V in solution.

## 4. Conclusions

In this study, the formation of Fe/V bearing MTA from the impurities-bearing TiOSO_4_ solution was investigated, with the following conclusions:

(1) Fe^3+^ and V^3+^ easily hydrolyzed with TiO^2+^ and entered the MTA lattice, resulting in these impurities being difficult to remove by washing. Traditionally manipulating Ti^3+^ could only remove one of Fe or V.
(2) Based on thermodynamic calculations, the conditions for neither Fe nor V hydrolysis were determined: (a) Ti^3+^ = 0.01 M, *F* = 3.0, T = 130 °C. (b) Ti^3+^ = 0.01 M, *F* = 3.5, T = 150 °C.
(3) To improve the Ti hydrolysis ratio (>0.95) and reduce the reaction time (4–6 h), two-step hydrolysis was provided (130 °C, 2 h + 150 °C, 2 h, Ti^3+^ = 0.01 M, *F* = 3.0), and impurity levels of Fe/V were notably reduced (Fe = 21 ppm, V = 145 ppm).
(4) The residual V impurity may have been due to the adsorption of the V^3+^-SO_4_^2-^ complex on the surface of the MTA particles.

These findings will provide new insights into the control of the hydrolysis of impurity ions in solutions and help to optimize the process of making TiO_2_ pigment from TBFS.

## Figures and Tables

**Table 1:** The composition of TBFS determined by XRF.

Elements	TiO_2_	CaO	SiO_2_	MgO	Al_2_O_3_	TFe	MnO	V_2_O_5_
wt.%	21.4	27.2	27.0	8.8	12.8	1.5	0.7	0.2

**Table 2:** The composition of impurity bearing TiOSO_4_ solution determined by ICP-AES and titration.

Component	Ti	Mg	Al	Fe	Mn	V	*F*-Value	Ti^3+^
mol·L^−1^	0.719	0.520	0.722	0.027	0.013	0.006	3.02	0.011

**Table 3:** The theoretical pH at the beginning of the impurity ions hydrolysis.

Ions	Mg^2+^	Al^3+^	Fe^2+^	Fe^3+^	V^3+^	VO^2+^	Mn^2+^
mol·L^−1^	0.520	0.722	0.029	0.029	0.005	0.005	0.013
p*K*_sp_(25 °C) [16]	10.74	32.88	15.1	37.4	34.4	22.13	12.73
Precipitation pH	8.74	3.17	7.22	2.05	3.30	7.39	10.39

**Table 4:** The impurity content (TiO_2_ wt.%) of Fe-MTA and V-MTA.

	TiO_2_ (%)	SO_3_ (%)	Mg (ppm)	Al (ppm)	Fe (ppm)	V (ppm)	Mn (ppm)
Fe-MTA	79.2	3.16	<5	1138	3854	<4	<4
V-MTA	78.4	3.31	<5	1202	8	1205	<4

**Table 5:** The analysis of the anatase (101) peak in XRD patterns.

	2θ (°)	Peak Height	FWHM	Crystal Size (Å)
Blank	25.223	5799	0.617	134
Fe-MTA	25.186	4377	0.821	100
V-MTA	25.352	4825	0.805	102

**Table 6:** The conc. of free H_2_SO_4_ for different *F*-value and TiOSO_4_ conc.

Conc. (mol·L^−1^)	Impurities-Bearing TiOSO_4_ Solution				Sulfate Process
TiOSO_4_	0.719				2.375
*F*-value	1.5	2.0	2.5	3.0	1.8
Free H_2_SO_4_	0.163	0.459	0.755	1.051	1.115

## Data Availability

Data are contained within the article.
